# Prevalence of ADHD Symptoms amonge Male Preschoolers Based on Different Informants in Ahvas City of Iran

**DOI:** 10.5402/2011/709653

**Published:** 2011-10-25

**Authors:** Ashraf Tashakori, Razieh Afkandeh

**Affiliations:** Ahvaz Jundishapur University of Medical Sciences, Golestan Avenue, 418 Gelayol Alley, Ostadan Street, Ahvaz 61357 33118, Iran

## Abstract

*Background*. This cross-sectional study evaluated rate of reported ADHD symptoms among male preschoolers in Ahvaz by different informants and rate of their agreement. *Method*. One hundred ninety-two 5-6-year old boys from nine kindergartens in Ahvaz were selected. Teacher and parents' Conners ADHD rating scales were used. *Findings*. For 133 children, questionnaires were returned. Prevalence of children with positive questionnaire was 24.06%, with only parent questionnaire was 4.51%, with only teacher questionnaire was 17.29%, and with both informants was 2.25%. Teacher-parent agreement was low (*P* = 0.0001). *Conclusions*. ADHD symptoms are frequently reported by informants and may be more reported by teachers than parents and teacher-parent agreement may be low. Interview with different informants and observation of child behavior are needed for documentation of diagnosis.

## 1. Introduction

Attention Deficit Hyperactivity Disorder is a developmental disorder with persistent pattern of distractibility, hyperactivity, restlessness, and other disturbances of executive function [1, 2]. It is one of the most prevalent psychiatric disorders in childhood that is estimated to occur in 2–19% of children in the world [3, 4] and 14.2% among school-aged children in Ahvaz [5]. There is a variation in prevalence of ADHD depending on different informants. Because the reports of informants are the basic tools for diagnosis of ADHD, so clinician must rely on parents' or teachers' subjective opinion. Also, acceptable behaviors are different by attitude; therefore prevalence of ADHD is varied [6, 7]. Boys are more affected than girls with the ratio ranging from 2 : 1 to 9 : 1 [1, 3, 4]. ADHD is one of the costly public health problems because of its significant impairment in all important areas of functioning in different age groups as discussed elsewhere [8–10]. The course of symptoms from infancy to about the age of 3 years is variable, because of temperamental differences. By the age of 4 years, the diagnosis is more persistent into middle childhood [11, 12]. Although its onset is usually before the age of 7 years, there is fewer data about prevalence of this disorder in preschoolers [3, 13, 14]. Prevalence of ADHD in preschoolers is between 2% and 19.3% [1, 13, 15–18]. Recent literature emphasizes cognitive, relational, and behavioral problems of preschoolers with ADHD [2, 8, 10, 12, 14, 19, 20]. Early diagnosis can provide early intervention and diminish negative impact of the disorder [1]. In a systematic review about the prevalence of ADHD in Iran, most studies had been conducted on the population of primary-school-aged children. However, only 3 studies had been conducted on preschoolers [13]. According to the above-mentioned subjects this study evaluated rate of reported ADHD symptoms among male preschoolers in Ahvaz by different informants and rate of their agreement.

## 2. Materials and Methods

This study was a part of the other study that we carried out to evaluate growth parameters of ADHD children and institutional review board of Ahvaz Jundishapur University of Medical Sciences (AJUMS) approved it. This descriptive cross-sectional study determined prevalence of ADHD symptoms in male preschool-aged children in kindergartens (preschoolers had been admitted by both school and kindergarten in Ahvaz during our study) of Ahvaz (Ahvaz is an urban area with partial well income) city in Iran and estimated rate of agreement between parents and teachers. Nine kindergartens were selected from different areas of Ahvaz by cluster sampling in 2010. One hundred ninety-two 5-6-year old boys were selected. Boys were selected because of the higher prevalence of ADHD in male [1, 3, 4] and better comparison between reports of parents and teachers. After the consent of teacher and parents, they were asked to complete a Conners rating scale for each of boys. Score ≥15 was considered positive for ADHD. Score of 15–20 was considered as mild, 21–25 as moderate, and 26–30 as severe. This scale consists of ten items for assessment of hyperactivity and inattention. Keith Conner developed this highly abbreviated version of Connors rating scale for use by parents and teachers in 1973 [21]. It is sensitive (>90%) and specific (77% to 98%), and its correct classification rate is 84% to 96% [22]. According to discriminant validity and good sensitivity, it is an effective and standard measure of ADHD [23]. Also, it is broadly used in Iranian population for screening of ADHD [13]. For descriptive analyses, prevalence and incidence were determined. Kappa coefficient was used for measure of agreement between parents and teachers.

## 3. Results

For 133 children questionnaires were returned, but for other children questionnaires were not returned. Prevalence and incidence of children with and without positive Conners rating scale by different informants were determined (Table 1). Of 32 children with positive rating scale 22 (67.75%) children had mild, 9 (28.13%) children had moderate, and 1 (3.13%) child had severe ADHD symptoms. Nine parents' Connors rating scale and 26 teachers' Connors rating scale were positive. For 3 children both parents and teachers' Conners rating scales were positive. Kappa coefficient did not show agreement between parents and teachers (*P* = 0.0001).

## 4. Discussion

In our study the prevalence of reported ADHD symptoms among male preschoolers in Ahvaz by informants was 24.6% among 5-6 year-old boys in kindergartens in Ahvaz. This prevalence rate is higher than in previous studies. The prevalence of ADHD in Mashhad city, in northeast of Iran, in male preschoolers was 18.1% [18]. One study in India found prevalence of ADHD in male preschoolers around 19.3% [16]. These studies had used Conners rating scale and parent interview. Parent interview may determine functional impairment or differentiation of other diagnosis. The reported prevalence based on functional impairment has been lower than that based on symptom alone [3].

 The prevalence of ADHD in studies is different depending on the diagnostic criteria and tools used for evaluation of ADHD [3, 4]. The diagnosis of ADHD in young children is a challenging problem, because differentiation of cardinal symptoms of ADHD from temperamental characteristics and routine daily behaviors of preschoolers is very difficult. So both overdiagnosis and underdiagnosis may occur. 

On the other hand, there is some controversy in the literature whether ADHD is a biological disorder or cultural disorder. A review study suggested that cultural differences are one of the factors that in studies affect the prevalence of ADHD [4]. Because reports of informants are basic tools for diagnosis of ADHD and acceptable behaviors are different by attitude, therefore ADHD may be cultural dependent. Other review studies suggested that ADHD is not a cultural construct and there is not any difference between prevalence in different countries or cultures while all studies use the same criteria [6].

In our study most of the children with positive rating scales had been only reported by teachers and a fewer number of them had been only reported by parents or by both parents and teachers. This result suggests that ADHD symptoms may be more reported by teachers than parents and teacher-parent agreement may be low. Other literature also reported that there is a variation in prevalence of ADHD depending on different informants [3, 4]. For example, a review study reported higher prevalence of ADHD based on teacher reports than parent reports and lowest rate when using combined teacher and parent assessment [3]. Other study in epileptic children concluded that identifying children as ADHD more likely occurs by parent report than teacher report and agreement is highest for more severe ADHD symptoms [24]. Also, in our study rate of severe ADHD was lower than mild and moderate ADHD. This may be due to abstention from admission of severe ADHD children by teachers or parents. If severe ADHD children had been available, teacher-parent agreement would have become higher, a result similar to study in epileptic children [24]. Also, one study suggested that ADHD is more common in one setting rather than two setting screening studies [4].

Informant disagreement may be due to several factors: different contexts of the observation, informant psychopathology, situation, kind of activity, informant attitude, and their characteristics. Children with ADHD may differently act in each setting. They are more hyperactive in unstructured situation. Conversely, problems in attention are most often seen in classroom setting and carrying out tasks. So rule-enforced activity may be a differentiating factor.

Children with ADHD have better function in one-to-one versus group situation. Also, they may better interact with animal and younger or older person than themselves, because of unconditional love in these relationships [7, 22, 25]. Reasons of high prevalence of ADHD based on teacher report may be label bias due to training specific to ADHD [26], teachers' perception of difficult behavior of youngest children in the classroom [27] that sometimes contains children with one year age difference.

One limitation of this study was lack of cooperativeness of some parents and teachers that did not refer questionnaires. Besides, severe ADHD children were not available.

## 5. Conclusion

In conclusion, our study suggested that ADHD symptoms are frequently reported by teachers and parents by means of screening questionnaire. Although some false positive may exist in our results, nonetheless, high score in Connors rating scale represents complaint and difficulties of teachers and parents that must be addressed. Participation of parents and teachers of children with positive screening test in interview session and observation of child behavior are needed for documentation of diagnosis.

## Figures and Tables

**Table 1:** The prevalence and incidence of children with and without positive rating scale for ADHD by different informants.

	*n*	%
Without positive rating scale	101	75.93
With positive rating scale	32	24.06
Only parent questionnaire	6	4.51
Only teacher questionnaire	23	17.29
Both parent and teacher questionnaire	3	2.25
Total	133	100
